# Your Morals Depend on Language

**DOI:** 10.1371/journal.pone.0094842

**Published:** 2014-04-23

**Authors:** Albert Costa, Alice Foucart, Sayuri Hayakawa, Melina Aparici, Jose Apesteguia, Joy Heafner, Boaz Keysar

**Affiliations:** 1 Center of Brain and Cognition, CBC, Universitat Pompeu Fabra, Barcelona, Spain; 2 Institució Catalana de Recerca i Estudis Avançats (ICREA), Barcelona, Spain; 3 Department of Psychology, University of Chicago, Chicago, Illinois, United States of America; 4 Departament de Psicologia Bàsica, Evolutiva i de l’Educació, Universitat Autònoma de Barcelona, Barcelona, Spain; 5 Department of Economics, Universitat Pompeu Fabra, Barcelona, Spain; 6 Department of Human Development and Family Studies, University of Connecticut, Storrs, Connecticut, United States of America; University of Buenos Aires, Argentina

## Abstract

Should you sacrifice one man to save five? Whatever your answer, it should not depend on whether you were asked the question in your native language or a foreign tongue so long as you understood the problem. And yet here we report evidence that people using a foreign language make substantially more utilitarian decisions when faced with such moral dilemmas. We argue that this stems from the reduced emotional response elicited by the foreign language, consequently reducing the impact of intuitive emotional concerns. In general, we suggest that the increased psychological distance of using a foreign language induces utilitarianism. This shows that moral judgments can be heavily affected by an orthogonal property to moral principles, and importantly, one that is relevant to hundreds of millions of individuals on a daily basis.

## Introduction

People often believe that moral judgments about “right” and “wrong” are the result of deep, thoughtful principles and should therefore be consistent and unaffected by irrelevant aspects of a moral dilemma. For instance, as long as one understands a moral dilemma, its resolution should not depend on whether it is presented in a native language or in a foreign language. Here we report evidence that people tend to make systematically different judgments when they face a moral dilemma in a foreign language than in their native language.

According to some models of moral psychology, moral judgment is driven by a complex interaction of at least two forces: intuitive “automatic” processes prompted by the emotional content of a given dilemma, and rational, effortful, controlled processes driven by the conscious evaluation of the potential outcomes [1]–[3]. In this dual process account, intuitive processes generally support judgments that favor the essential rights of a person (deontological judgments), while rational controlled processes seem to support judgments favoring the greater good (utilitarian judgments), regardless of whether or not they violate an individual’s rights [4]–[11]. The relative weight of intuitive and rational processes in moral judgments can vary, and lead to more or less deontological or utilitarian judgments. As such, establishing which conditions favor each of these two mechanisms is fundamental to understanding the psychology of morality (for a review, see [12]). The present study explores whether using a foreign language, as hundreds of millions of individuals do every day, can have a systematic impact on these processes.

There are good reasons to expect that using a foreign language would reduce utilitarian resolutions of moral dilemmas. For example, there is evidence that utilitarian choice relies on controlled processes that require cognitive resources, and that an increase of cognitive load [6] or stress [13], [14] reduces utilitarian choice in moral dilemmas. The added cognitive load and anxiety of using a foreign language could therefore reduce the use of controlled processes and subsequently reduce utilitarian choice. That is, to the extent that utilitarian choice reveals a higher contribution of controlled processes and such processes require the recruitment of cognitive resources, then conditions that increase cognitive load such as the use of a foreign language should decrease utilitarian choice.

Despite this potential impact of cognitive load, we propose that using a foreign language results in the opposite, that it actually increases utilitarian choice. In general, a foreign language elicits less intense emotional reactions relative to a native language [15]–[18]. For example, skin conductance responses as well as the perceived force of emotional phrases are reduced when presented in a foreign language compared to a native language [19]. Additionally, heuristic biases that are driven by emotional factors, such as loss aversion, are reduced when people make decisions in a foreign language [20], [21]. Such reduced emotionality, we argue, promotes a more reasoned, controlled process that leads to a utilitarian choice.

Hence, we hypothesize that moral judgments in a foreign language would be less affected by the emotional reactivity elicited by a dilemma. This hypothesis makes a clear prediction: when faced with moral dilemmas in a foreign language, utilitarian judgments should be more common than in a native language. We tested this prediction in two experiments using the well-known *trolley dilemma* [22].

## Experiment 1

We used the “footbridge” version of the trolley dilemma [23], where one imagines standing on a footbridge overlooking a train track. A small on-coming train is about to kill five people and the only way to stop it is to push a heavy man off the footbridge in front of the train. This will kill him, but save the five people. A utilitarian analysis dictates sacrificing one to save five; but this would violate the moral prohibition against killing, and imagining physically pushing the man is emotionally difficult and therefore people routinely avoid that [6], [24]. If we are correct, then people would be more likely to opt for sacrificing one man to save five when dealing with such moral dilemmas in a foreign language than in their native tongue.

### Method

#### Participants

We collected data from several native/foreign language populations: English/Spanish (N = 112) in the US, Korean/English (N = 80) in Korea, English/French (N = 107) in France, and Spanish or English/Hebrew (N = 18) in Israel. The native language varied in Israel because we recruited participants in a school for learning Hebrew. Participants were late learners of the foreign language who did not grow up speaking it at home. Sufficient proficiency to understand the instructions was assessed through comprehension checks. Proficiency and background information are included in Table 1. Participation was voluntary and the experimental protocol was approved by the IRB of the Social Sciences Division of the University of Chicago. Seventy-two additional participants were excluded because they either failed to comprehend the scenario (N = 41), grew up with the language (N = 16), did not clearly indicate an answer (N = 3), or were not native speakers of the native language (N = 12).

**Table 1:** Experiment 1′s participants’ details.

**Percentage Female**	53%
**Mean age**	21 yrs
**Mean age of FL acquisition**	14 yrs
**Self-rated proficiency in the foreign language**	
***(1 = least fluent, 5 = most fluent)***	
**Written comprehension**	3.1
**Written production**	2.8
**Oral production**	2.6
**Oral comprehension**	2.9

The specific age at time of experiment and self-rated proficiency was not collected for the French sample. However, all of these participants were undergraduate students whose ages ranged from 18–23 at the time of the experiment.

#### Procedure and materials

All materials were translated from English and back-translated for comparability [25]. The consent form, materials and conversation with the experimenter were in the assigned language. Participants read a packet with the scenario and a cartoon depiction of the scene. After they indicated their decision, they answered questions regarding demographic and language background, and the foreign language packet contained a comprehension check. Crucially, within each language-pair group, participants were randomly assigned to either their native tongue (N = 158) or a foreign language (N = 159).

### Results and Discussion

Across all populations more participants selected the utilitarian choice, to save five by killing one, when using the foreign language than their native tongue (Table 2). The difference between the foreign and the native language condition ranged from 7.5 percentage points to 65 percentage points. Taking a weighted average across populations, we find that the rate of utilitarian decisions in a foreign language was increased by more than half compared to the native tongue (from 20% to 33%; χ^2^(1, N = 317) = 6.9, *p*<.01, φ = .148).

**Table 2:** Percentage of Utilitarian Decisions by Language Condition in Experiment 1.

Languages		Percent of utilitarian decisions	
Native	Foreign	Native	Foreign
Korean	English	0%	7.5%
English	Spanish	28%	44%
English/Spanish	Hebrew	10%	75%
English	French	20%	33%
**Weighted Average**		**20%**	**33%**

While for all four language groups in this experiment the pattern was in the predicted direction, we note two things that are worth considering. First, none of the Korean participants in the native language condition chose to push the man, which might seem unusual. This could reflect a cultural prohibition, and is consistent with the finding that East Asians are less likely to select the utilitarian choice with such dilemmas [26]. Despite this, the Korean group showed a 7.5 pp difference between the native and foreign language. The second thing to note is the unusually high difference for the group who used Hebrew as a foreign language. While the other three groups showed a modest difference between the native and foreign conditions of 7.5, 13, and 16 percentage points, that group showed a large 65 pp difference. Most likely this is an artifact of the small size of that group (N = 18) and should not be interpreted as reflecting any special quality of that group. To make sure our results are not determined by this group we re-analyzed the data without it and found the same pattern. Of people using their native language, 21% made the utilitarian decision as compared to 31% using a foreign language, χ^2^(1, N = 299) = 4.0, *p*<.05.

The results support the hypothesis that the reduced emotional resonance of a foreign language leads individuals to be less affected by an emotional aversion to pushing the man, allowing them to make more utilitarian decisions. Experiment 2 replicated the effect and evaluated two alternative explanations.

## Experiment 2

We considered the following two alternative explanations to Experiment 1. First, because a foreign language is more difficult, participants are more likely to respond at random. Given that only 20% of the participants made the utilitarian choice when using a native tongue, occasional random responding would push the proportion upward towards 50%. If this is true, then our findings are not due to reduced emotional reactions but to a response pattern. To evaluate this, we included a version of the trolley dilemma that is much less emotional [22]. In this “switch” dilemma, the trolley is headed towards the five men, but you can switch it to another track where it would kill only one man. People are more willing to sacrifice the one man by pulling the switch than by pushing him off the footbridge, and one of the primary reasons is that pulling the switch is less emotionally aversive [5]. If reduced emotional reactions determine our effect, we should not find an effect of language in the less emotional switch dilemma. If it is random responding, we should find a reversed effect, as random response should push utilitarian choice down towards 50%.

A second alternative explanation assumes that people might be more utilitarian not because of the language per-se but because of cultural norms. For example, Spanish-speaking societies tend to be more collectivistic than English speaking societies [27], [28]. If using Spanish primes such norms, it could lead one to prefer the common good over the rights of individuals. This could have led participants in Experiment 1 who used Spanish as a foreign language to push the man to his death more, not because of the foreign-ness of the language but because of its associated norms. The multitude of the native/foreign language pairs we used makes this alternative less likely, but it is important to evaluate it directly. Experiment 2 did so by crossing language and native-ness, using both Spanish/English and English/Spanish populations.

### Method

#### Participants

Data from 725 participants are included in the analyses, including 397 native speakers of Spanish with English as a foreign language, and 328 native speakers of English with Spanish as a foreign language. The study was conducted in classrooms. Agreement from the teachers and students to conduct the study was obtained prior to the day of the study. In the classroom, participants were verbally informed about the study. It was emphasized that participation was voluntary and anonymous, and that participation could be aborted at any time. The study was part of a project approved by the ethics committee (Comité Etic d’Investigacio Clinica, Parc de Salut Mar, Barcelona), who also waived the need for written informed consent from the participants. Participants provided their verbal informed consent and freely decided to take part in the study or not. If they decided to take part, the only personal information they were required to provide was their age, gender, and native language. Native Spanish participants who had spent more than 10 months in an English speaking country were excluded. At the end of each problem, participants were asked to rate their understanding of the problem (regarding language); those who rated it less than 50% were excluded from the study. Thus, all participants included in these studies had a moderate level of proficiency in their foreign language. Participants’ background and proficiency information are provided in Table 3.

**Table 3:** Experiment 2′s participants’ details.

	Native Spanish Speakers (N = 197)	Native English Speakers (N = 168)
**Percentage Female**	71%	73%
**Mean age**	21 yrs (range 18–33)	21 yrs (range 18–28)
**Mean age of FL acquisition**	8.4 yrs	12 yrs
**Months Immersed in the foreign language country**	1.1 mths	2.8 mths
**Self-rated proficiency in the foreign language ** ***(1 = least fluent, 7 = most fluent)***		
**Written comprehension**	5.3	4.9
**Written production**	4.6	4.6
**Oral production**	4.2	4.1
**Oral comprehension**	5.3	5.1
**Self-rated understanding of the problem**	87.3%	84.7%

#### Materials

The materials included the footbridge and the switch dilemmas but no pictures. Each participant received two dilemmas, the footbridge and the switch dilemmas, with the order counterbalanced across participants.

#### Procedure

The study was conducted in classrooms of 10 to 50 students with various backgrounds (e.g., psychology, neuroscience, criminology, linguistics, media, architecture, education). Participants received the instructions and then the two dilemmas either in their native language or the foreign one. All students within each classroom performed the task in the same language. The order of presentation was counter-balanced across participants. It was emphasized that there was no incorrect answer and that the choice was personal. The experimenter stayed in the classroom during the whole session, which lasted about 10 minutes.

### Results and Discussion

**Figure 1:**
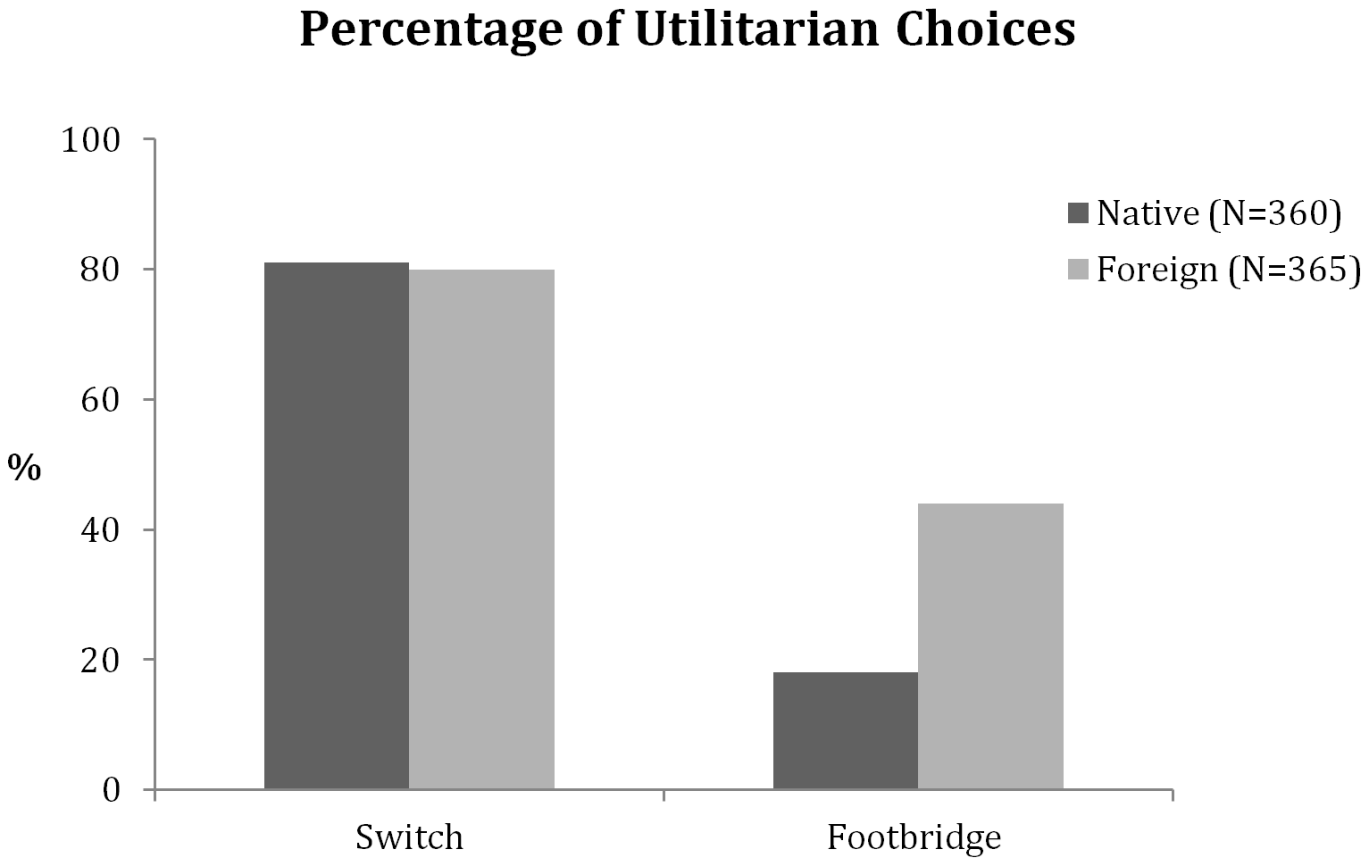
Percentage of utilitarian decisions (Experiment 2). Percentage of utilitarian decisions for the two versions of the trolley problem in the native language condition and the foreign language condition.

We analyzed the choices for the footbridge and the switch problem separately. For the footbridge dilemma, participants’ choices were strikingly different depending on the native-ness of the language (Figure 1). While only 18% of the participants decided to push the man to his death when using their native tongue, fully 44% of them chose to push him when using a foreign language, *χ* ^2^ (1, N = 725) = 57.3, *p*<.001. These results replicate the results of Experiment 1 and show an even larger difference, from a 13 percentage-point increase in utilitarian choices in Experiment 1, to 26 percentage points in Experiment 2.

#### Reduced emotionality or random responding?

**Figure 2:**
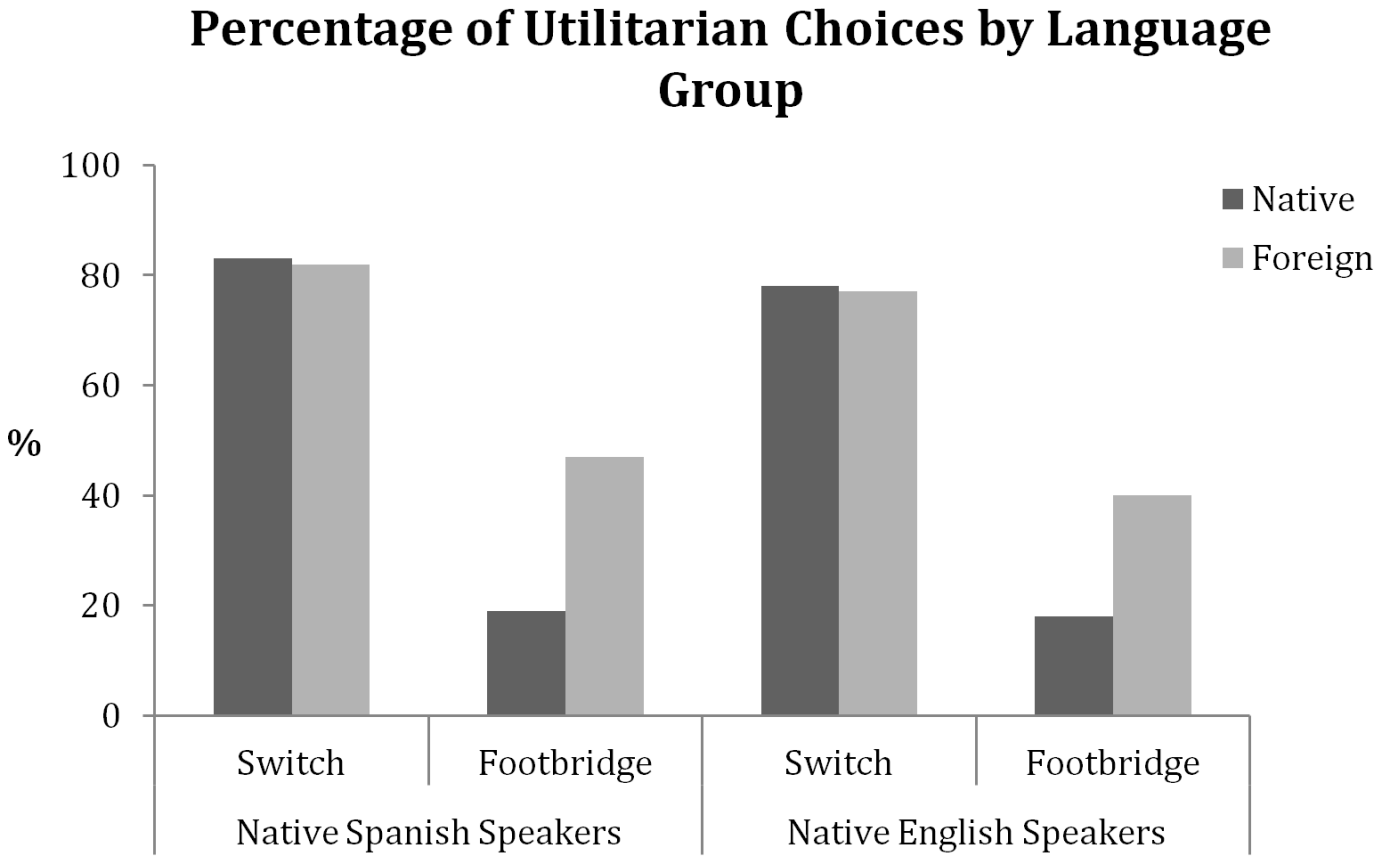
Percentage of utilitarian decisions by language group (Experiment 2). Percentage of utilitarian decisions for the two versions of the trolley problem in the native language condition and the foreign language condition, divided by native language group. Native Spanish speakers using Spanish (N = 200) or English (N = 197); native English speakers using English (N = 168) or Spanish (N = 160).

Recall that the switch dilemma is much less emotional and that in general people predominantly choose the utilitarian option. Indeed, our participants preferred to divert the train, killing one person to save five, both in their native (81%) and foreign (80%) language (*χ^2^*(1, N = 725) = 0.03; *p* = .85; Figure 1). This was true with English or Spanish as the foreign language (Figure 2). So while a foreign language increased utilitarian choices with the emotional footbridge dilemma, it did not have an effect on the “colder” switch dilemma. This is consistent with our assumption that a foreign language increases utilitarianism by increasing emotional distance, but given the high level of utilitarian choices it could also reflect a ceiling effect.

More importantly, these results allow us to evaluate whether a foreign language simply increases the tendency to respond randomly. While the results for the footbridge dilemma are consistent with this account, the results of the switch dilemma contradict it. Given the 81% rate of utilitarian choice with the native language in the switch dilemma, random responding would predict a *reduction* in utilitarian choice with a foreign language. There is no hint of such a reduction, strongly arguing against this alternative explanation.

#### Evaluating a cultural explanation

Crossing the languages in Experiment 2 allows us to evaluate the second alternative explanation for the results of Experiment 1, which suggested that the willingness to sacrifice the man depended on the culture that is associated with the language, not on the native-ness of the language. The results from Experiment 2 do not support this claim as the effect of language for the footbridge dilemma was independent of the native tongue of the participants and of the culture associated with the language of the session. Participants made more utilitarian choices in Spanish (40%) than English (18%) when Spanish was foreign (*χ* ^2^ (1, N = 328) = 20.90, *p*<.0001), but more utilitarian decisions in English (47%) than Spanish (19%) when English was foreign (*χ* ^2^ (1, N = 397) = 37.14, *p*<.0001; Figure 2). This pattern clearly contradicts a cultural explanation to our findings.

**Figure 3:**
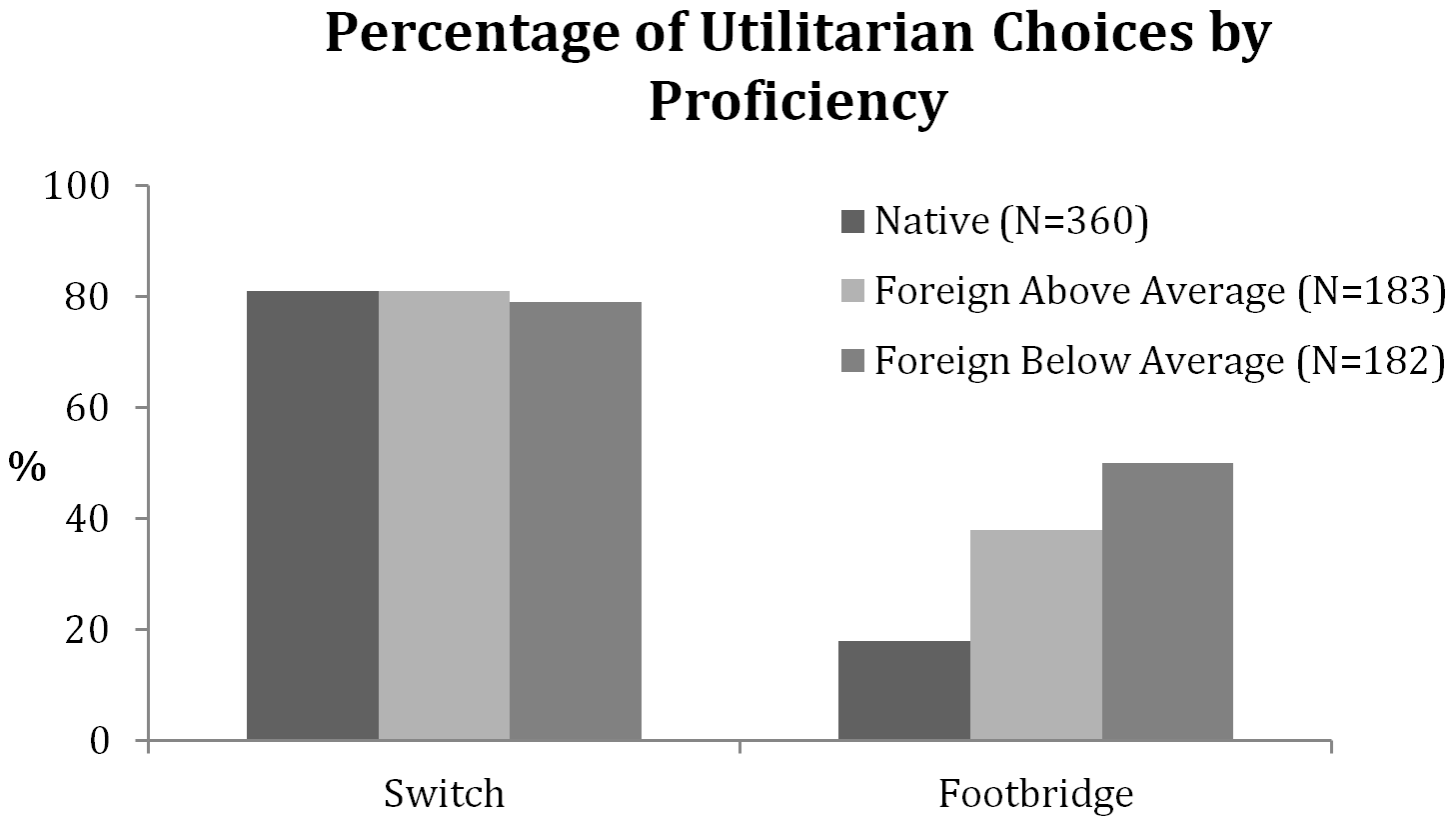
Percentage of utilitarian decisions by proficiency (Experiment 2). Percentage of utilitarian decisions for the two versions of the trolley problem in the native language condition and the foreign language condition, divided by self-rated proficiency level.

With increased proficiency, a foreign language could become more emotionally grounded [15]. We conducted a post-hoc analysis, splitting participants according to their self-rated proficiency level as either above-average or below-average. The pattern suggested that the increase in utilitarian judgments for the more emotional footbridge dilemma depends somewhat on the proficiency in the foreign language. The increment in utilitarian responses in a foreign language, although present for both proficiency groups, was larger for lower (32 percentage-points) than for higher proficiency participants (20 percentage-points; see Figure 3). The difference between the lower and the higher proficiency groups was significant (*χ* ^2^ (1, N = 365) = 5.11, *p*<.02).

A potential caveat when interpreting these results is that participants might not have properly understood the text in a foreign language. This is unlikely because the effect of the foreign language differed between the switch and footbridge problems, and participants reported having a good understanding of the problems. More importantly, a subgroup of participants (N = 237 for foreign; N = 218 for native) also received the Cognitive Reflection Test (CRT; [29]), a test of logical reasoning composed of three problems. Among these participants, those using a foreign language actually outperformed those using a native language in logical reasoning, with 60% and 47% of participants providing at least one correct answer out of three, respectively. Therefore, we are confident that participants’ level of proficiency was sufficient for full understanding of the text, and that the results are due to the emotional distance that the foreign language provides rather than lack of comprehension.

## General Discussion

We have shown that people’s moral judgments and decisions depend on the native-ness of the language in which a dilemma is presented, becoming more utilitarian in a foreign language. These results are important for models of moral decision making because they show that identical dilemmas may elicit different moral judgements depending on a seemingly irrelevant aspect such as the native-ness of the language. Most likely, a foreign language reduces emotional reactivity, promoting cost-benefit considerations, leading to an increase in utilitarian judgments.

The reduction of the emotionality elicited by a foreign language may promote psychological distance in general. Increasing psychological distance leads individuals to construe situations in more abstract terms, which in some circumstances aligns with more utilitarian decision making [30], [31]. For instance, a more abstract mind-set is associated with a greater focus on ends than means, leading to more utilitarian decisions in moral dilemmas like the footbridge problem [32].

Another factor that may contribute to the effect of a foreign language on moral judgement is cognitive fluency. Studies have shown that disrupting cognitive fluency or slowing down decisions decreases decision biases by moving individuals to a more careful and deliberative mode of processing [33], [34]. Given that using a foreign language could reduce cognitive fluency [35], [36], it might diminish the impact of intuitive processes on moral judgment. That said, our results suggest that the emotional reaction has an impact above and beyond it. According to the logic of the CRT, the worse the performance is on the task, the more people are using intuitive rather than controlled processes. For individuals who performed the worst on the CRT task and solved none of the three problems, using a foreign language increased utilitarian choices by 27 percentage points. This increase was virtually identical to the overall impact of a foreign language (26 percentage points). This suggests that the effect persists for moral judgments even when the foreign language is not disfluent enough to disrupt intuitive problem solving as indicated by the CRT.

Note, however, that we did find an effect of language proficiency on the percentage of utilitarian choices in Experiment 2. That is, the more proficient the participants considered themselves in the foreign language the more their decision patterns resembled that of the native speakers. In our view, this result suggests that increasing foreign language proficiency may promote emotional grounding, hence eliciting similar emotional reactions to that of a native language. Future studies could evaluate this interpretation as it makes a clear prediction that highly proficient foreign language speakers should show a markedly reduced foreign language effect on moral judgments.

All the accounts above have in common the notion that moral dilemmas faced in a foreign language may promote deliberative processes and reduce emotionally-driven responses. Hence, they fit very well with models of moral decision making that consider moral judgments as the result of the interplay of intuitive emotionally driven processes and rational thoughtful processes [1]–[3]. The results are also consistent with the notion that in some cases decision making in a foreign language could be less affected by intuitive heuristics.

This discovery has important consequences for our globalized world as many individuals make moral judgments in both native and foreign languages. Immigrants face personal moral dilemmas in a foreign language on a daily basis, sometimes dilemmas with even larger stakes such as when serving as a jury member in a trial. Foreign languages are used in international, multilingual forums such as the United Nations, the European Union, large investment firms and international corporations in general. Moral choices within these domains can be explained better, and are made more predictable by our discovery. Indeed, awareness of the impact of the native-ness of the language on moral dilemmas is fundamental to making more informed choices. Whether you believe that adherence to moral rules is a better choice or that a utilitarian cost-benefit analysis is the better one [12], [37], regardless of your morals, your decisions should not be a function of the native-ness of the language you are using. It shouldn’t matter if you are considering the life of “the large man” or of “el hombre grande.” But it does matter. Given that what we have discovered is surprising and unintuitive, increasing awareness of the impact of using a foreign language may help us check our decision-making context and make choices that are based on the things that should really matter.
